# Arsenic uptake by *Agrostis capillaris*, as related to its genotypic diversity in the area of historical ore mining and processing

**DOI:** 10.1038/s41598-024-63830-1

**Published:** 2024-06-12

**Authors:** Agnieszka Dradrach, Kamila Nowosad, Bartosz Kozak, Anna Karczewska

**Affiliations:** 1 Institute of Agroecology and Plant Production, Wrocław University of Environmental and Life Sciences, Wrocław, Poland; 2 Department of Genetics, Plant Breeding and Seed Production, Wrocław University of Environmental and Life Sciences, Wrocław, Poland; 3 Institute of Soil Science, Plant Nutrition and Environmental Protection, Wrocław University of Environmental and Life Sciences, Wrocław, Poland

**Keywords:** Soil, Arsenic, Common bentgrass, Bioaccumulation, Translocation, Ecotypes, DNA, Ecology, Plant sciences, Biogeochemistry, Environmental sciences

## Abstract

Common bentgrass *Agrostis capillaris* L. is known as tolerant to toxic elements. A hypothesis was examined that its ecotypes growing in historically polluted sites show a limited arsenic uptake and have genetic features that distinguish them from commercially available cultivars. The study was conducted in Złoty Stok, a historical area of arsenic mining. Additionally, two commercial cultivars were grown in pots with arsenic-rich soils. Based on arsenic concentrations in plant roots and shoots, bioconcentration and translocation factors BCF and TF were calculated. Commercial cultivars indicated many times higher BCF shoots and TF values compared to field plants. DNA analysis of leaf blades showed a clear distinction between the plants growing in some sites and patches in the field, and also a gene overlap between the plants in the field and commercial forms. The research did not allow for identification of ecotypes with exceptionally limited arsenic uptake. Moreover, there were no significant differences between the genotypic characteristics of plants growing in polluted sites and those poorly tolerant grown from commercially available seeds. Apparently, other factors, and not genetically determined features, are responsible for *A. capillaris* tolerance to arsenic in Złoty Stok.

## Introduction

Arsenic (As) is a highly toxic element to biota^1–3^. Significant soil contamination with arsenic is primarily associated with former ore mining and processing areas, such as Złoty Stok in Poland, where arsenic concentrations in soils and mine dump materials can reach thousands of mg kg^−1^^4–6^. Such soil contamination poses serious environmental risks, including those caused by possible uptake of this element by plants that constitute food for humans and both wild and farmed animal consumers. Effective limiting of As uptake by plants and its input into food chains is of key importance from the point of view of environmental risk^7–9^. It should be stressed that the concept of risk analysis has become recently the main approach used for the assessment of land pollution and decisions related to the need of remediation measures^10–12^. One of the grass species commonly found in arsenic-contaminated areas is the common bentgrass *Agrostis capillaris* L.^4,13–17^. This species constitutes an important part of plant communities in polluted areas and is known for its significant tolerance to high concentrations of toxic elements in soil^18–23^.

This research tested the hypothesis that different ecotypes of *A. capillaris*, including those from the former arsenic mining area in Złoty Stok, vary in their tolerance and ability to uptake arsenic from soils and translocate it to aboveground parts. We also made an attempt to check whether tolerant ecotypes from the field differ in terms of genetic features from commercially available ones. A review of literature indicated that tolerant ecotypes of various plant species may develop in contaminated sites, implementing a strategy of As avoidance and limited translocation from roots to shoots^24–27^. The occurrence of such ecotypes in the sites of historical ore mining in England was already described several decades ago by Macnair^28,29^. Research by various authors confirmed that some plant species growing on soils very rich in As have the ability of As avoidance^21,30–33^, although Nandillon et al.^17^ pointed out that the differences between tolerant and intolerant ecotypes do not have to be reflected in differences in arsenic root-to-shoot translocation. It has already been proven that various mechanisms may be responsible for plant tolerance towards As. Reactions in the rhizosphere, particularly those involving soil microbial activity, can significantly influence plant tolerance to arsenic by affecting its chemical speciation, adsorption, solubility, bioavailability, mobility, and soil–plant transfer. The decrease or increase in As phytoavalability is usually attributed to the microbially induced redox transformations of As between As(V) and As(III)^34,35^. Thiol-reactive cysteine-rich peptides such as phytochelatins and metallothioneins, that can be produced both by plants and by rhizospheric fungi and bacteria, strongly bind with As and convert it to a non-toxic forms^15,36–38^. In response to stress caused by arsenic, plants also can evolve physiological defense mechanisms, such as the production of antioxidant enzymes, including superoxide dismutase, catalase or glutathione, as well as induced synthesis of phytochelatin^39^. Overexpression of phytochelatin synthase genes can increase plant tolerance to As-stress^40^. It should be emphasized that the tolerance of ecotypes exposed to As may also be genetically conditioned and inherited^16,28,29^. Tolerant and intolerant ecotypes of plants may show differences in genome-wide expression and variation in nucleotide sequences^41,42^. Many transcription factors associated with different gene families are involved in coordinating arsenic responsive genes, contributing to stress signaling, arsenic-toxicity and arsenic-tolerance. The presence of such genes was confirmed by bioinformatic analyses in various studies, however their identification is complex and yet practically impossible^43–45^. Therefore, molecular data in research should be closely combined with analysis of phenotypic data.

This study was aimed at analyzing As accumulation by the ecotypes of *A. capillaris* growing in different As-enriched sites in the area of Złoty Stok. The parameters of As uptake by plants were related to genotypic diversity of those ecotypes. The research assumed that the ecotypes of *A. capillaris* that have developed in historically polluted sites may differ in their response to soil As, which should be reflected in parameters that characterize As uptake and bioaccumulation. The following parameters were considered: bioaccumulation factor BAF, defined as the ratio of As concentration in plant biomass to total As concentration in soil, bioconcentration factor BCF: the ratio of As concentration in plant biomass to the concentration of easily soluble forms of As in the soil^4,14,46–49^ and translocation factor TF, i.e. the ratio of As concentrations in the aboveground parts parts of plants to those in plant roots. An attempt was made to identify As-tolerant ecotypes of *A. capillaris*, growing in the field and having low BAF, BCF and TF values. Possible genotypic differences between these tolerant ecotypes and commercial forms of *A. capillaris* available as seed were looked for. The practical aim of the research was to identify such genotypes that are tolerant and capable of implementing the As avoidance strategy, so that the concentrations of arsenic in plant shoots will not exceed 4 mg kg^−1^, which is, in accordance with the EU Directive (2002)^50^, the upper safe limit for feed and a related limit value in ecological risk assessment^8,9^.

## Materials and Methods

The research included field work and laboratory analyses, as well as a mini-pot vegetation experiment. Field samples were collected in June 2022, and the pot experiment was carried out in May–July 2022.

### Field research

**Figure 1:**
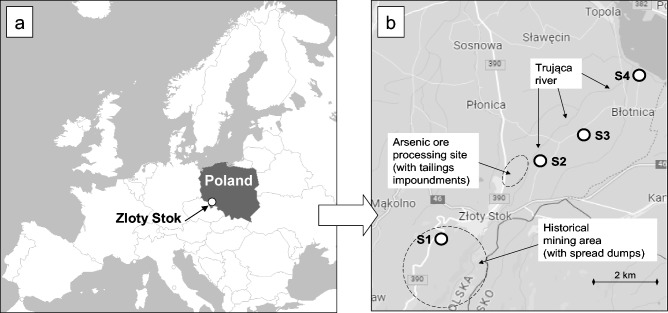
Location of study sites. (**a**) Złoty Stok in the map of Europe, (**b**) study sites S1–S4 in Złoty Stok.

Populations of *A. capillaris* growing in four sites (S1–S4) in the area historically enriched in arsenic in Złoty Stok were selected for field studies (Fig. 1).

Site S1 represents a mining dump, while sites S2, S3, and S4 are grasslands used as hay meadows or wastelands. They all were located in the Trująca stream valley. It is worth mentioning that the stream name is a Polish word meaning “poisonous”, which is related to water contamination with arsenic. A detailed description of these sites is presented in Table 1 and in Supplementary Materials (Table S1, Fig. S1).

**Table 1:** Characteristics of study sites.

No	Altitude m a.s.l	Site description	Usage
S1	480–490	A mine dump with an area of 2.4 ha, partly reclaimed by covering with approx. 10 cm of productive humus-rich soil. The origin of this soil has not been established. The area is legally protected due to inhabitation by a large population of *Orchis mascula* L.^4,49^	Ecological area
S2	270–280	A fresh meadow, in the past repeatedly flooded with the water of the Trująca stream as well as by tailings released from nearby tailings impoundments^5^	Hay meadow
S3	250–260	The meadow located approximately 5 m above the stream level. The enrichment in arsenic is here mainly of geochemical nature, most likely as a halo effect associated with gold ores^51^. Wind-borne arsenic-rich particles can also contribute to environmental contamination in this site	Hay meadow
S4	220–230	A meadow, partly covered with trees, in the floodplain of the Trująca stream, near its mouth into the artificial Topola dam reservoir on the Nysa Kłodzka river	Wasteland

In each site, three patches of *A. capillaris* (P1-P3), situated 10–50 m apart from each other, were identified and chosen for the study. Within each patch, plant samples and soil samples (from a depth of 0–15 cm) were collected for analysis. The aboveground parts of grass were harvested in the field by cutting directly above the crowns. Soil lumps with grass roots were transported to the laboratory, where the roots were carefully separated from soil. Plant material was washed with tap water and distilled water. Then, soil and plant material was air-dried and prepared for chemical analysis. Material collected from each patch was divided into three subsamples, so that the analyses were performed in triplicates. Moreover, typical leaf blades of *A. capillaris* were collected from all patches under study (10 samples from each patch) for DNA analyses. The samples were placed in test tubes, transported to the laboratory in liquid nitrogen, and stored frozen.

Additionally, extra amount of soil material, representative for each site, was collected from the close neighborhoods of *A. capillaris* patches (ca. 10 kgfrom each site) and prepared for mini-pot experiments (S1–S4-Pot) in parallel with As-spiked soils.

### Mini-pot experiment

Apart from plant samples collected from the field, research material included also the plants grown from two commercially available forms of *A. capillaris* seeds in mini-pots (with a volume of 1.2 dm^3^). The experiment was carried out with soil material collected from all the sites in the field (S1–S4-Pot) and with a loamy soil, spiked with arsenic (50, 100, 200, 500 mg kg^−1^ As) in the form of sodium arsenate Na_3_AsO_4_^.^12H_2_O, adjusted to pH 6.2), and stabilized at constant moisture for 1 month, which ensured effective As ageing and immobilization^52^. The mini-pot experiment was carried out in three replicates. The commercial forms of *A. capillaris* (obtained from Weberseeds, NL) were: wild one (hereinafter referred to as D1) and a breeding cultivar (referred to as D2). Grass seeds did not germinate in soils spiked with 500 mg kg^−1^ As; hence, this concentration was excluded from further analysis. In the first design of the experiment, it was also planned to include seeds to be collected from the field in Złoty Stok. Unfortunately, the attempts to obtain well-germinating seeds were unsuccessful and did not allow for a parallel pot experiment with a native material. Plants grew in mini-pots for 2 months. In that time grass shoots were pruned and discarded twice to obtain a better tillering effect. Thereafter, both the above-ground parts and grass roots were collected, dried, and weighted. Soil samples were taken and prepared for analysis, similarly to the material brought from the field. Leaf blades were collected for DNA analyses.

### Analysis of soil properties

Stone and gravel fractions were removed from soil samples and their contribution to soil volume was roughly determined. Basic properties of fine soil were analyzed, which involved determination of soil texture, the content of organic carbon and total nitrogen, soil pH and cation exchange capacity CEC. All these analyses were carried out using standard methods^53,54^. More specific soil examination focused on total concentrations of As and its extractability. The total (or precisely: pseudototal) concentrations of arsenic in soils were determined after microwave (CEM-MARS Xpres) digestion in aqua regia. Easily soluble species of As, often considered as currently bioavailable^55^, were extracted from soils with 1 M NH_4_NO_3_ (ISO 19730). The concentrations of As in all the digests and soil extracts were measured by ICP-AES, on the iCAP 7400 instrument, Thermo Scientific. Analytical methods were validated with appropriate CRMs. Determination of total As in soil samples was validated with the CRMs certified for aqua-regia-extracted elements (CNS 392 and CRM 027).

### Chemical analysis of plant material

Chemical analysis of plant material involved determination of total As concentrations in the aboveground biomass and in plant roots. The concentrations of As in oven-dried (50 °C, 48 h) and ground plant samples were determined after microwave (CEM-MARS Xpres) digestion in concentrated nitric acid HNO_3_, preceded by oxidation with 30% perhydrol^4,49^. The concentrations of As in the digests were determined on the ICP-AES instrument, as in the case of soil analyses. The analytical method was validated using two plant CRMs (BCR-414 and DC-73349). Additionally, in particular in the case of very low concentrations of As in analytes, the results obtained with ICP-AES were randomly controlled by ICP-MS (8800 Triple Quad, Agilent). Arsenic bioaccumulation (BAF), bioconcentration (BCF), and translocation factors (TF) were calculated based on arsenic concentrations in soils, roots, and shoots, following the equations detailed by Dradrach et al.^4^.

### Analysis of plant DNA

The genetic structures of populations growing in various localities in the field were characterized based on the analysis of DNA extracted from plant leaves and the examination of PCR molecular markers^56–59^. Samples of soil and plant material from pot experiments were subject to analogous analyses. Leaf fragments were preserved in liquid nitrogen. DNA isolation was performed using Genomic Midi AX Plant kits produced by A&A Biotechnology. A set of 100 primers generating ISSR (Inter Simple Sequence Repeat) markers was used to analyze the differentiation. The PCR reaction was performed on 10 randomly selected objects^60^. After electrophoretic separation on the Qiaxcel device, the primers that allowed for the demonstration of polymorphism between the tested objects were selected for further analyses, and the obtained results were transformed into a binary matrix, on the basis of which the genetic distance was calculated according to the Nei'79 formula^56^. Genetic diversity was presented in the form of a dendrogram using the UPGMA algorithm^61^.

### Statistics

Parameters characterizing soil properties and As uptake from soil by plants (BAF, BCF and TF) were compared within individual sites S1-S4 and between the sites. The least statistically significant differences between the sites and experimental treatment in the pot experiment were calculated by Fisher’s test, at *p* < 0.05, using Statistica 13.0. The R statistical package, with additional libraries such as agegenety, poper, hirstat, and pegasus, was used for data analysis^62,63^. The primary analysis focused on calculating Nei's genetic distance using the genotyping matrix^56^. This was followed by the construction of a dendrogram using the UPGMA method. Additionally, Principal Component Analysis (PCA) was performed based on the genotype matrix to explore the genetic structure of the population. The PCA results were visualized using R integrated with ggplot tools.

## Results

### Soil properties

The soils in the studied sites differed in their properties. The variability of basic soil properties within the sites was relatively small and mainly associated with differences in organic matter content (Table 2). The soils on the mine dump (S1) had a high share of skeletal parts (40–50%), and their earthy parts had a sandy loam (LS) texture. The soils of sites S2 and S4 in the Trująca stream valley had also a sandy loam texture, therefore the soil with a similar texture was selected for mini-pot experiments. In the site S3, the soil within the P1 patch differed significantly from P2 and P3, both in terms of texture (loamy sand LS vs. silty loam SiL) and pH values. In general, the soils were mostly acidic or slightly acidic, with the pH in the range of 4.6–6.2, except for the patch P1 in site S3, mentioned above, where soil was neutral, with pH 6.9 (Table 2).

**Table 2:** Soil properties.

Site	Patch	Percentage of skeletal parts, %	Texture of fine soils				Corg g kg^−1^	N g kg^−1^	pH 1 M KCl	CEC cmol ^+^ kg^−1^
			Percentage of fraction, mm			Textural group				
			0.5–2.0	0.002–0.5	< 0.002					
Field studies										
S1	P1	40	80	13	7	LS	133	7.4	5.1	15.2
	P2	50	75	22	3	LS	27	1.9	4.8	6.9
	P3	40	74	24	2	LS	61	3.5	5.0	5.9
S2	P1	5	57	36	7	SL	35	2.9	5.7	10.5
	P2	10	55	40	5	SL	68	4.8	6.2	8.9
	P3	3	61	31	8	SL	47	3.2	6.1	11.9
S3	P1	20	76	21	3	LS	12	0.9	6.9	5.5
	P2	15	40	52	8	SiL	29	2.0	4.9	13.6
	P3	10	42	51	7	SiL	14	1.0	4.8	12.2
S4	P1	< 1	56	31	13	SL	24	1.9	4.9	16.7
	P2	< 1	54	30	16	SL	29	2.8	4.6	16.9
	P3	< 1	68	24	8	SL	21	1.6	5.2	12.4
Pot experiments										
S1-Pot		25	83	14	3	LS	33	6.1	5.5	14.4
S2-Pot		11	53	41	6	SL	24	3.1	6.3	10.8
S3-Pot		3	53	45	2	SL	16	1.0	4.4	12.6
S4-Pot		< 1	59	33	8	SL	20	2.2	4.5	16.8
Spiked soil		< 1	72	18	10	SL	13	0.7	6.2	13.7

The texture and other properties of soil material collected from the field and used for the pot experiments did not differ considerably from corresponding soils brought to the laboratory together with grass roots (Table 2). Spiked soil had a texture of silty loam (SL), similar to that of most field-collected soils, however it was slightly poorer in organic matter (Table 2).

All soils collected from various sites in Złoty Stok had very high or high arsenic concentrations (Table 3), much higher than the geochemical background, estimated at 4.7 mg kg^−1^^3^. The average total As concentrations in the soils of objects S1–S4 were: 20,300; 3,550; 95 and 395 mg kg^−1^, respectively, while the concentrations of 1 M NH_4_NO_3_-extractable As were very low, with average values 11.3, 5.2, 0.7 and 0.3 mg kg^−1^, respectively, which constituted 0.06–0.67% of the total As.

**Table 3:** Arsenic in soils and the parameters of its accumulation in plants—field study.

Site	Patch	As in soil, mg kg^−1^			As in plants, mg kg^−1^		BAF	
		Total		Extracted with 1 M NH_4_NO_3_	Roots	Shoots	Roots	Shoots
		in patches	Mean ± SD in the site					
S1	P1	19,900	20,300 ± 5200	19.60	94.6	4.43	0.005	0.0002
	P2	25,700		7.70	2300	35.9	0.089	0.0014
	P3	15,300		6.62	82.1	3.37	0.005	0.0002
S2	P1	3110	3550 ± 1030	4.83	190	7.43	0.061	0.0024
	P2	4720		7.19	330	9.4	0.070	0.0020
	P3	2810		3.56	270	11.9	0.096	0.0042
S3	P1	135	95 ± 39	1.60	7.8	1.3	0.058	0.0096
	P2	69		0.30	83.6	16.7	1.212	0.24
	P3	82		0.31	67.1	10.7	0.818	0.13
S4	P1	339	395 ± 119	0.18	85.5	3.2	0.252	0.0094
	P2	312		0.20	93.9	5.4	0.301	0.0173
	P3	530		0.53	42.5	1.2	0.080	0.0023

The concentrations of As and its extractability in soils brought from Złoty Stok and used in the pot experiments (S1-S4-Pot soils) did not differ considerably from soils sampled from corresponding *A. capillaries* patches, and fell within the standard deviation ranges calculated for each site (Table 4). In the soils spiked with As, the share of 1 M NH_4_NO_3_-extractable As was slightly larger than in Złoty Stok soils, i.e. 0.9–1.5% of the total As.

**Table 4:** Arsenic in soils and its accumulation in plants—pot experiment. Mean data of 3 replicates.

Soil	*A. capillaris*- form	As in soil, mg kg^−1^		Dry mass of shoots g /pot	As in plants, mg kg^−1^		BAF	
		Total	Extracted with 1 M NH_4_NO_3_		Roots	Shoots	Roots	Shoots
S1-Pot	Breeding *A.c.* form D2	19,600	3.61	0	No growth	n.d	n.d	
S2-Pot		4020	7.32	0.39	1940	113	0.483	0.028
S3-Pot		133	0.19	1.98	106	24.5	0.797	0.184
S4-Pot		394	0.18	1.83	156	21.2	0.396	0.054
Spiked soil	Wild *A.c.* form D1	50	0.75	0.64	406	249	8.12	4.98
		100	0.92	0.40	792	340	7.92	3.40
		200	1.90	0.11	3050 ? ^a)^	2380 ?	15.3 ?	11.9 ?
	Breeding *A.c.* form D2	50	0.72	0.72	390	215	7.80	4.30
		100	0.98	0.49	450	325	4.50	3.25
		200	2.01	0.18	n.d	1220 ?	9.10 ?	6.10 ?

^a^Question marks indicate the data obtained from only one of three replicates, therefore the data should be treated as informative. At total As concentrations 200 mg kg^−1^, plants grew in pots very poorly. Root biomass of *A.c.* cultivar was not large enough for the analysis of As concentration, therefore it was not determined (n.d.)

### Arsenic uptake by plants

Observations and biomass data (Table 4) from the pot experiment indicated an adverse effect of high As concentrations on plant growth. Only single grass seedlings emerged and soon died on the S1-Pot soil, while the biomass of the shoots of plants growing on the S2-Pot soil was several times lower than that obtained on S3-Pot and S4-Pot soils. *A. capillaris* growing on spiked soil containing 200 mg kg^−1^ As had even lower biomass. However, the further discussion focused primarily on As concentrations in the roots and shoots of plants.

Chemical analysis of *A. capillaris* collected from all four field sites S1-S4 confirmed that generally the plants poorly took up arsenic, clearly demonstrating an avoidance strategy. The concentrations of As in plant roots were much higher than those in the aboveground parts of plants, which is typical for this element^3,25,26^, and was already reported in previous screening studies from the area of Złoty Stok^4,14^. The values of the root-to-shoot translocation coefficients (TF) were consequently very low, in particular in the most strongly contaminated sites S1, S2 and S4, where they did not exceed 0.06 (Table 3). However, in the site S3, they were significantly higher (0.16–0.20). Special attention should be drawn to the large differences between the patches in the S1 site, namely the P2 patch turned out to be quite different form P1 and P3 patches. These differences concerned both As concentrations in plant roots and in aboveground parts of plants. In the P2 patch, a very high concentration of As was found in *A. capillaris* roots, averaging 2300 mg kg^−1^ (Table 3), while the roots of this species in the patches P1 and P3 contained less than 100 mg kg^−1^ As. The concentrations of As in the shoots of *A. capillaris* growing in the P2 patch of the S1 site were also much higher than those in the P1 and P3 patches, amounting to 35.9 mg kg^−1^ and significantly exceeding the value of 4 mg kg^−1^ considered safe for consumers, including wild animals^50^. Arsenic concentrations in the shoots of grass in the P1 and P3 patches of the S1 site were considerably lower, i.e. 3.4 and 4.4 mg kg^−1^ on average, and those in *A. capillaris* growing in the other sites were in the range of 1.2–16.7 mg kg^−1^.

The concentrations of As in the biomass of plants grown form the commercial breeding cultivar D2 in the pot experiment, in soils from Złoty Stok (S2-, S3-, and S4-Pot), were significantly higher than those measured in plants collected from corresponding sites in the field (Table 4). This applied to both roots and particularly to the shoots. Obviously, the results of pot experiments cannot be directly compared with the results from the field^64–66^, but the differences between the ecotype of *A. capillaris* and the cultivar (breeding form) were apparently responsible for different As uptake and plant tolerance.

The grass grown in the spiked soil, took up As much more intensively (Table 4). There were no statistically significant differences between the D1 and D2 forms in this respect (Fisher’s test, *p* < 0.05). Arsenic concentrations in the shoots of commercial D1 and D2 grass were many times higher than those in plants growing in the field. At the total As concentration of 200 mg kg^−1^ in the spiked soil, grass emerged only in single replicates and took up extremely large amounts of As, above 1000 mg kg^−1^, in spite of the fact that the concentration of easily soluble As forms in soils, extracted with 1 M NH_4_NO_3,_, did not exceed 2.01 mg kg^−1^.

**Figure 2:**
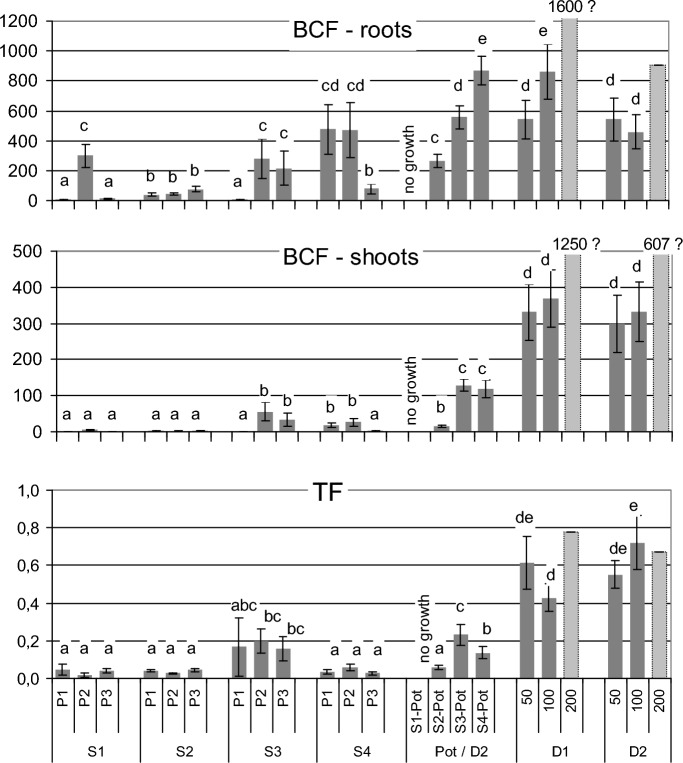
Comparison of BCF and TF parameters characterizing As uptake by *A.* *capillaris* in various sites in the field (S1–S4) and in a the pot experiments with the wild form (D1) and the breeding form (D2). Presented parameters: BCF roots: bioconcentration factor determined for roots, BCF shoots: bioconcentration factor determined for shoots, TF: translocation factor. Lowercase letters in the graphs indicate the groups that do not differ statistically significantly at *p* < 0.05. Bars marked with a dashed line illustrate data obtained based on a single value (as the amount of plant material in two of three replicates was insufficient for analysis).

The differences in As uptake by the grass that formed various patches in the field and in the pot experiment were reflected by strongly divergent BCF and TF values (Fig. 2). No statistically significant differences were found for BCF shoots and TF values within the highly enriched sites S1 and S2, and also S4. The TF values were in the S3 site significantly higher than those in S1, S2 and S4, while in the case of D1 and D2 grass grown in the spiked soil TF values were even higher (Fig. 2).

However, when considering the variability of BCF and TF parameters within the sites, it should be noted that the P2 patch differed considerably from the other two patches in the site S1, and P1 was different from the other two patches in the site S3 (Fig. 2). These differences should probably be attributed to different patterns of As uptake from soils by various ecotypes of *A. capillaries* that formed separated patches in those sites.

### Genetic diversity

**Figure 3:**
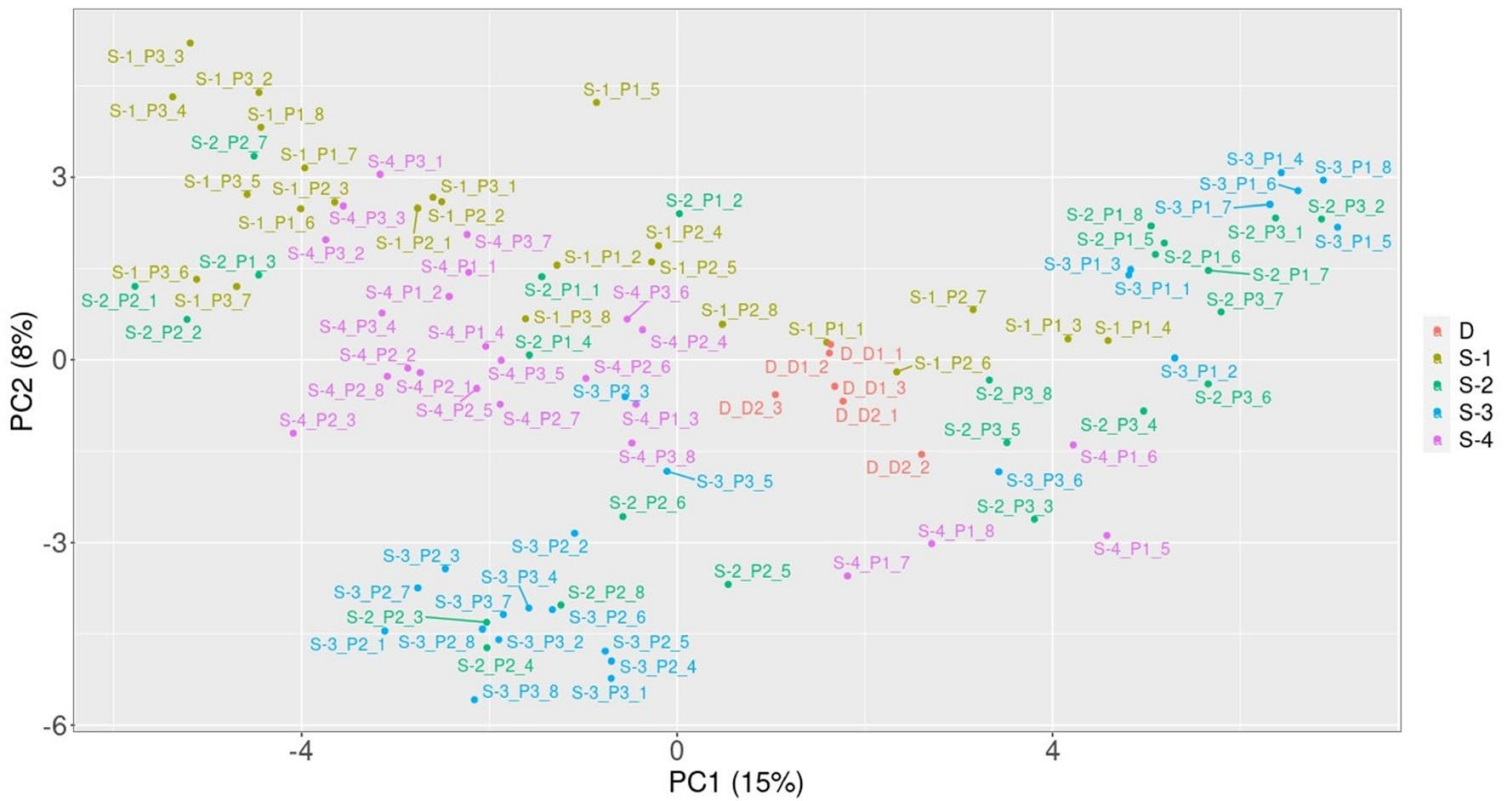
PCA map. Projection of principal components PC1 and PC2 onto the plane.

PCA analysis (Fig. 3) of the first two principal components suggests that *A. capillaris* populations at site S1 (Orchid Dump) are genetically diverse, with the P2 patch showing distinct genetic characteristics compared to the P1 and P3 patches. Similarly, plants growing at the site S3 differed in their DNA-related characteristics, in particular the P1 patch was clearly different from the other two patches. Both commercial forms of *A. capillaris*, referred to as 'wild' D1 and ‘breeding’ D2, showed high genetic similarity to each other. They also show significant similarity to *A. capillaries* growing in the site S4, as well as to some patches in the sites S2 and S3, and even in the site S1.

**Figure 4:**
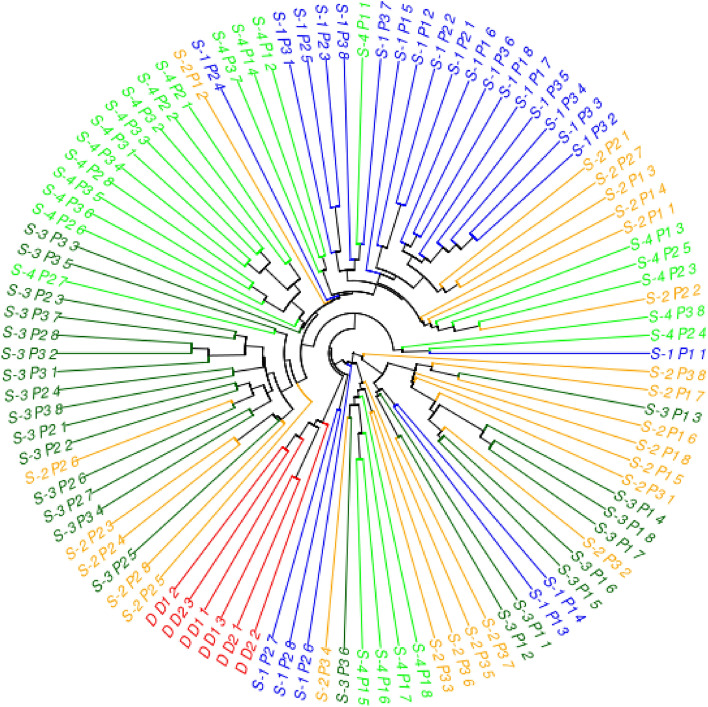
Dendrogram obtained by the UPGMA method based on the Nei’ genetic distance matrix.

These observations were also confirmed by the analysis of the dendrogram (Fig. 4). The dendrogram reinforces the clustering patterns observed in the PCA analysis, indicating that while there is clear genetic distinction between some patches, there is also a significant degree of genetic overlap between different sites, and in particular between the commercial forms (D1, D2) and those growing in the site S4. This suggests that there were potential genetic exchanges between these populations or that they shared common ancestral lines. Moreover, the genetic approximation of commercial forms to specific patches in different sites may indicate the wide spread of similar genetic varieties or the impact of human activity on the dissemination of these commercial forms of *A. capillaris*. Distinct clusters may be an indication of local adaptation. They may also suggest that different subspecies should be distinguished within *A. capillaris,* which may have important implications for conservation and management strategies of various populations of this grass.

## Discussion

Field results confirmed previous screening studies^4,14^, showing significant differences in arsenic concentrations in the aboveground biomass of *A. capillaris* in arsenic-enriched areas. The screening allowed to indentify single individuals of bentgrass with As concentrations in shoots over 110 mg kg^−1^, and those in roots up to 9400 mg kg^−1^^4,14^, which would possibly qualify this species as an As hyperaccumulator. Some authors have reported that *A. capillaris* can hyperaccumulate metal(loid) elements, including Pb and As, though this is controversial and questioned by others^18,67^. The present study failed to confirm that *A. capillaris* growing in the field can accumulate particularly high As concentrations in its shoots. The maximum As concentration found in the shoots of one replicate in the S1-P2 patch (detailed results not presented here) was 62.0 mg kg^−1^, while the mean for this patch was 35.9 mg kg^−1^ (Table 3). Arsenic concentrations in aboveground biomass are influenced by various factors, including the plant's developmental phase, the availability of phosphorus (an analogue of arsenic), and abiotic and biotic conditions in the rhizosphere^15,30,34,38,40,68^. Consequently, the plants collected for analysis at the same sites in the field, on different dates, may contain different concentrations of arsenic and other elements.

As mentioned above, our research confirmed that As concentrations in the biomass of *A. capillaris* within one site or even one patch can show great diversity, and as a consequence, the BAF and BCF coefficients can also vary considerably. The translocation factor (TF) appears to be a reliable parameter for characterizing the arsenic accumulation ability of plants within specific populations or ecotypes. A review of the knowledge regarding physiological mechanisms of As transport inside the plant indicates that the values of TF coefficient are determined, on the one hand, by the factors responsible for passive transport of elements, but on the other hand—by the ability to express genes that facilitate As transport (Pi) or, on the contrary, responsible for the secretion of As-immobilizing substances in the rhizosphere^36,41,43,69^.

Genetic diversity analysis did not reveal significant differences between *A. capillaris* ecotypes from Złoty Stok and commercially available forms, either wild or breeding, indicating their high genetic similarity. Similarly, small differences were also presented by Warnke and Barnaby^42^. Genotypic similarity of commercially available *A. capillaris* and plants collected at various locations in the polluted areas may indicate that all, or almost all, populations of *A. capillaris* occurring in the studied areas of Złoty Stok have a common primary origin, which could have been the sowing of meadows in the Trująca stream valley and other grasslands located nearby. Probably the top layer of soil from the Trująca stream valley (site S2) that constituted a seed bank, was used for the reclamation and revegetation of the Orchid Dump (site S1), located 4 km away, at over 200 m higher altitude (Fig. 1, Table 1). This fact explains also the occurrence of *Orchis mascula* on this dump. On the other hand, seed material from the site S2 was undoubtedly naturally carried to the site S4 (in the stream floodplain) and also to S3.

It should be noted, however, that the genetic characteristics of plants in different patches in the field showed considerable differences, which coincided with the differences in As uptake parameters. The plants in the P1 and P3 patches on the Orchid Dump (S1) had particularly low BAF and BCF values, both for roots and aboveground parts (Table 3, Fig. 2), and the analysis of their DNA profiles (Figs. 3 and 4) indicated specific features that distinguish them from other populations examined. Similarly, the genotypic characteristics of the P1 patch in the S3 site are different from those of P2 and P3 patches in that site. The plants that formed this patch showed very low BCF values (both roots and shoots). To some extent, it may be due to the particularly high soil pH value. It is known that soil pH is important for development of various mechanisms responsible for plant tolerance towards As. For example, acidic conditions support the synthesis of As-binding phytochelatins^34^. However, it is possible that the mechanisms of natural selection and adaptation of plants to pH conditions allowed for the establishment there of the ecotype with specific features, different from those of the plants in the P2 and P3 patches.

Our research showed that specific features and mechanisms have developed in the populations of *A. capillaris* growing in the Złoty Stok mining area, that are involved in limiting arsenic uptake by these plants and their tolerance to high As concentrations in soil. On the contrary, the plants grown in pot experiments from commercially obtained seeds take As much more intensively, in particular in the case of As-spiked soil.

Plants grown in pot experiments from the same D2 commercially obtained seeds, sown into soils brought from Złoty Stok, had in fact intermediate parameters of As-uptake in relation to those collected from As mining areas and those growing on spiked soils. This seems to support the fact that various factors, directly related to soil, in particular the soil microbiome, have a significant impact on *A. capillaris* tolerance and As uptake. However, the fact that in the pot experiment in the soil taken from the Orchid Dump (S1-Pot) the grasses did not grow at all, and in the soil from the Trująca valley (S2-Pot) their growth was very poor, indicates the importance of other factors, probably genetically determined, that are responsible for the tolerance of *A. capillaris* ecotypes towards As in these most highly enriched places. It has already been proven for instance that arbuscular mycorrhizal fungi (AMF) can strongly affect the uptake, displacement, and speciation of As in plants. Various tools are involved in those effects including limited uptake of As into plant roots by affecting the protein synthesis of As and P-uptake channels, increased uptake of phosphate, As absorption by AMF hyphae, secretion of glomalin-related soil proteins and other mechanisms^27,70^.

Future research should focus on: (1) Identifying particularly tolerant ecotypes through more detailed genetic analysis; (2) Conducting advanced genetic studies to identify transcriptionally active regions related to arsenic tolerance mechanisms; (3) Investigating biotic and abiotic rhizosphere factors affecting plant tolerance to arsenic; (4) Acquiring and examining seeds from various sites in Złoty Stok to study generative reproduction and tolerance in pot experiments. What is interesting is to what extent these plants reproduce from seeds, and to what extent they reproduce generatively, through runners overwintering in the soil. The issue of *A. capillaris* tolerance to high As concentrations in soils in the area of the former As mining and processing plant in Złoty Stok requires detailed analysis.

## Summary and conclusions

This study confirmed that *A. capillaris* is a relatively As-tolerant species, capable of As avoidance, which may be useful for phytostabilization. The research did not allow, however, for clear identification of specific ecotypes of *A. capillaris* that would have exceptional tolerance to high concentrations of As in soils and strongly limited uptake of this element from soils. However, it was confirmed that there are some differences in genotypic characteristics of plants, which can be related to their ability to efficiently reduce arsenic uptake from soils. The research also confirmed that plants grown from commercially available seeds do not have sufficient tolerance to arsenic, even though their genotypic features do not significantly differ from those of *A. capillaris* growing in polluted sites in the mine area of Złoty Stok. The study proved that the tolerance of *A. capillaris* towards As and its uptake from soils depend on numerous other factors, and to some extent are conditioned by the characteristics of the soil rhizosphere, which require more comprehensive research.

### Supplementary Information

Supplementary Information.

## Data Availability

In this research, anonymous presence-absence variant polymorphism markers were used, specifically Inter-Simple Sequence Repeat (ISSR) markers. Polymorphic bands obtained on gels did not reveal chromosomal positions of the variants. These limitations associated with ISSR markers made it impossible to generate the data necessary for the standard formats used by genetic databases. The data supporting the findings of this study are available upon reasonable request. In particular, the dataset for genotypic matrix is available as compiled into an Excel file. To obtain the available data from this study, please contact either the corresponding author, A. Karczewska (anna.karczewska@upwr.edu.pl) or the second author, K. Nowosad (kamila.nowosad@upwr.edu.pl).

## Abbreviations

BAF: Bioaccumulation factor
BCF: Bioconcentration factor
TF: Translocation factor
CRMs: Certified reference materials
PCA: Principal component analysis
UPGMA: Unweighted Pair-Group Method using arithmetic Averages
