# Supplementary material for: Arsenic uptake by Agrostis capillaris, as related to its genotypic diversity in the area of historical ore mining and processing
Source: Sci Rep. 2024 Jun 12;14:13488. doi: 10.1038/s41598-024-63830-1 (PMC11169496; doi:10.1038/s41598-024-63830-1)
Supplement: Supplementary file 1 — Supplementary Information. [file 41598_2024_63830_MOESM1_ESM.pdf]

Supplementary Materials

Arsenic uptake by *Agrostis capillaris*, as related to its genotypic diversity in the area of historical ore mining and processing

Agnieszka Dradrach , Kamila Nowosad , Bartosz Kozak , Anna Karczewska *

* Corresponding Autor: anna.karczewska@upwr.edu.pl

Location of study sites

GPS coordinates provide the detailed location of study sites S1-S4 and the patches P1-P3 (Table S1). Additionally, the photos (Fig. S1) show the study sites S1-S4.

Table S1. GPS coordinates for study sites

Site No	Patch No	GPS coordinates [1]
S1	P1	50.437994, 16.855006
	P2	50.438076, 16.855234
	P3	50.438381, 16.855373
S2	P1	50.458357, 16.894478
	P2	50.458480, 16.893642
	P3	50.457742, 16.894049
S3	P1	50.465238, 16.907847
	P2	50.464760, 16.908898
	P3	50.464446, 16.908748
S4	P1	50.485425, 16.935515
	P2	50.485302, 16.935354
	P3	50.485377, 16.935901

[1] GPS coordinates indicate a central point of each patch

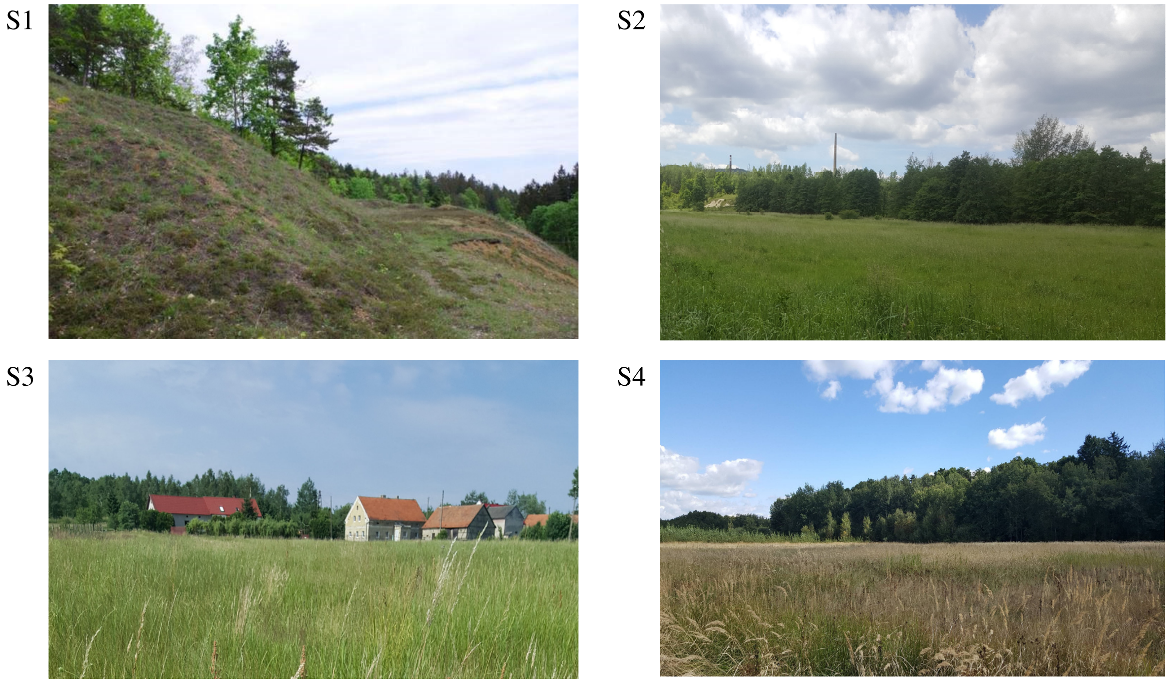

Fig. S1. General view of the study sites S1-S4.

Identification of *A. capillaris* species

In this study, any particular herbarium resources were not used to identify the species. Therefore, no information has been provided regarding the voucher specimens and the voucher numbers. The species was identified based on the grass identification guides [71-74].

Below, there is a description of *A. capillaris*, extracted from CABI compendium [74].

"*A. capillaris* is a low-growing, rhizomatous, perennial grass that forms dense swards of quite fine leaves that taper almost directly from the ligule down to the finely pointed tip. The flowering panicle is finely branched with numerous very small spikelets forming a reddish-purple haze over the mat of leaves. Culms are tufted, geniculate or decumbent and rooting at base, 20-70 (occasionally 100) cm tall. Leaf sheaths are smooth with linear blades, flat or inrolled, 2-15 cm x 1-4 mm, scabrous or nearly smooth with acuminate apex. The ligule on non-flowering shoots is 1-2 mm, shorter than wide and truncate. The panicle is elliptic in outline, up to 20 cm long, open and very lax with 2-5 spreading branches per node. Branches of the panicle are capillary, 1.5-3.5 cm, with purplish brown spikelets (1.5-2.5mm). Glumes are elliptic-lanceolate, subequal or lower glume slightly longer, lower glume scabrid along keel, the upper glume often smooth with acute apex. The entire plant is hairless. Seeds are small and brown; roots have scaly rhizomes and occasionally stolons glume often smooth with acute apex. The entire plant is hairless. Seeds are small and brown; roots have scaly rhizomes and occasionally stolons."

References to Supplementary Materials

71. Kozłowski, S. *Trawy - właściwości, występowanie i wykorzystanie.* Powszechne Wydawnictwo Rolnicze i Leśne, Poznań (in Polish), pp. 145-147 (2012).

72. Rutkowski, L. *Klucz do oznaczania roślin naczyniowych Polski niżowej.* Wydawnictwo Naukowe PWN, Warszawa (in Polish), pp. 595-597 (2022).

73. CABI compendium. https://www.cabidigitallibrary.org/doi/10.1079/cabicompendium.3830. *Accessed 28 May, 2024.*

74. Pladias. Database of Czech flora and vegetation. https://pladias.cz/taxon/overview/Agrostis%20capillaris. *Accessed 28 May, 2024.*
